# Disseminating Metaproteomic Informatics Capabilities and Knowledge Using the Galaxy-P Framework

**DOI:** 10.3390/proteomes6010007

**Published:** 2018-01-31

**Authors:** Clemens Blank, Caleb Easterly, Bjoern Gruening, James Johnson, Carolin A. Kolmeder, Praveen Kumar, Damon May, Subina Mehta, Bart Mesuere, Zachary Brown, Joshua E. Elias, W. Judson Hervey, Thomas McGowan, Thilo Muth, Brook L. Nunn, Joel Rudney, Alessandro Tanca, Timothy J. Griffin, Pratik D. Jagtap

**Affiliations:** 1 Bioinformatics Group, Department of Computer Science, University of Freiburg, 79110 Freiburg im Breisgau, Germany; blankclemens@gmail.com (C.B.); gruening@informatik.uni-freiburg.de (B.G.); 2 Department of Biochemistry, Molecular Biology and Biophysics, University of Minnesota, Minneapolis, MN 55455, USA; easte080@umn.edu (C.E.); kumar207@umn.edu (P.K.); smehta@umn.edu (S.M.); brow4261@umn.edu (Z.B.); tgriffin@umn.edu (T.J.G.); 3 Minnesota Supercomputing Institute, University of Minnesota, Minneapolis, MN 55455, USA; jj@umn.edu (J.J.); mcgo0092@umn.edu (T.M.); 4 Institute of Biotechnology, University of Helsinki, 00014 Helsinki, Finland; carolin.kolmeder@helsinki.fi; 5 Department of Genome Sciences, University of Washington, Seattle, WA 98195, USA; damonmay@uw.edu (D.M.); brookh@uw.edu (B.L.N.); 6 Computational Biology Group, Ghent University, Krijgslaan 281, B-9000 Ghent, Belgium; Bart.Mesuere@ugent.be; 7 Department of Chemical & Systems Biology, Stanford University, Stanford, CA 94305, USA; josh.elias@stanford.edu; 8 Center for Bio/Molecular Science & Engineering, Naval Research Laboratory, Washington, DC 20375, USA; Judson.Hervey@nrl.navy.mil; 9 Bioinformatics Unit (MF1), Department for Methods Development and Research Infrastructure, Robert Koch Institute, 13353 Berlin, Germany; MuthT@rki.de; 10 Department of Diagnostic and Biological Sciences, University of Minnesota, Minneapolis, MN 55455, USA; jrudney@umn.edu; 11 Porto Conte Ricerche Science and Technology Park of Sardinia, 07041 Alghero, Italy; tanca@portocontericerche.it

**Keywords:** metaproteomics, functional microbiome, bioinformatics, software workflow development, Galaxy platform, mass spectrometry, community development

## Abstract

The impact of microbial communities, also known as the microbiome, on human health and the environment is receiving increased attention. Studying translated gene products (proteins) and comparing metaproteomic profiles may elucidate how microbiomes respond to specific environmental stimuli, and interact with host organisms. Characterizing proteins expressed by a complex microbiome and interpreting their functional signature requires sophisticated informatics tools and workflows tailored to metaproteomics. Additionally, there is a need to disseminate these informatics resources to researchers undertaking metaproteomic studies, who could use them to make new and important discoveries in microbiome research. The Galaxy for proteomics platform (Galaxy-P) offers an open source, web-based bioinformatics platform for disseminating metaproteomics software and workflows. Within this platform, we have developed easily-accessible and documented metaproteomic software tools and workflows aimed at training researchers in their operation and disseminating the tools for more widespread use. The modular workflows encompass the core requirements of metaproteomic informatics: (a) database generation; (b) peptide spectral matching; (c) taxonomic analysis and (d) functional analysis. Much of the software available via the Galaxy-P platform was selected, packaged and deployed through an online metaproteomics “Contribution Fest“ undertaken by a unique consortium of expert software developers and users from the metaproteomics research community, who have co-authored this manuscript. These resources are documented on GitHub and freely available through the Galaxy Toolshed, as well as a publicly accessible metaproteomics gateway Galaxy instance. These documented workflows are well suited for the training of novice metaproteomics researchers, through online resources such as the Galaxy Training Network, as well as hands-on training workshops. Here, we describe the metaproteomics tools available within these Galaxy-based resources, as well as the process by which they were selected and implemented in our community-based work. We hope this description will increase access to and utilization of metaproteomics tools, as well as offer a framework for continued community-based development and dissemination of cutting edge metaproteomics software.

## 1. Introduction

Microbiome research has offered promising insights into microbial contributions to human health [1] and environmental dynamics [2]. Microbiome responses can be studied by a variety of approaches, including genome and transcriptome sequencing (metagenomics and metatranscriptomics, respectively), protein expression profiling (metaproteomics), and metabolite characterization (metabolomics). Over the years, the metagenomics-based approach has been the major approach for most microbiome studies, mainly because of the advances in sequencing technology [3] and development of statistical and analytical tools [4].

Recent trends in microbiome research have shown the promise of other “omic” approaches, with metaproteomics receiving much attention as an approach with great promise as a complement to more mature metagenomics approaches [5,6]. Metaproteomic studies identify the proteins that are actively being expressed by a community of microbiota under specific conditions [7]. Researchers have been promoting the potential benefits of metaproteomics for a better understanding of microbiome dynamics—particularly since it can provide insights into the functional state of the microbial community, beyond what can just be predicted by metagenomics [6,8].

Although the metaproteomics approach has been used for more than a decade, it is still emerging and has not yet become an approach routinely utilized by the microbiome research community. This has been primarily due to the technical difficulties associated with the approach. However, with recent advances in sample preparation, improved sensitivity of protein detection by mass spectrometry (MS), and new informatics tools for data analysis and interpretation, more researchers are turning to metaproteomics and realizing its potential in microbiome research [9].

Metaproteomics research holds promise in its ability to offer mechanistic insights into microbiome activity by performing functional analysis on identified peptides and proteins [10]. For example, microbiome studies have shown that the suite of metabolic pathways within microbiota from different persons tends to remain relatively consistent, even though microbial taxa may display considerable variation between individuals [11]. 

**Figure 1:**
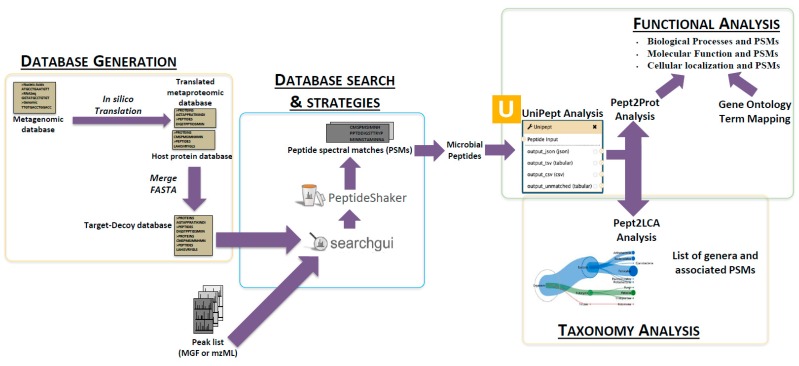
Generalized metaproteomics schema: Identification of metaproteome peptides is a complex workflow consisting of metaproteome sequence database generation (in FAST-ALL (FASTA) format) and peak processing of tandem mass spectrometry (MS/MS) data (in Mascot Generic Format (MGF) of mzML format). These two output files are used to match observed MS/MS spectra to predicted peptide sequences. This generates a list of bacterial peptide–spectral matches (PSMs). Later, the bacterial PSMs can be parsed out and subjected to functional analysis and taxonomic analysis for biological insight.

One of the key areas of advancement in metaproteomics over the past decade lies within the branch of informatics. New approaches continue to emerge across all the core areas of metaproteomics informatics, which include: (a) protein sequence database generation methods for microbial communities [12,13,14,15,16]; (b) database search methods for matching tandem mass spectrometry (MS/MS) data to peptide sequences [17,18]; and (c) interpretation methods and tools for taxonomic and functional analysis (Figure 1) [19,20,21].

Despite these many advances, metaproteomic informatics remains very much a work in progress because of many unresolved challenges. Unlike single-organism proteomics, the protein sequence FAST-All (FASTA) databases for metaproteomics, which contain the predicted proteomes of multiple organisms, can be extremely large and complex [18]. It is not uncommon for the in silico translation of metagenome assemblies to a predicted metaproteome to contain hundreds of thousands to millions of predicted protein sequence entries. To reduce the possibility of mis-assigned spectra, it is common practice to include a FASTA-formatted host database and common laboratory contaminant proteins (e.g., skin keratins, proteases). For example, the study of the human oral microbiome would contain human epithelial cells proteins as part of the host database, in addition to microbial proteins from the consortia that form dental plaque. Algorithms and strategies for matching MS/MS to peptide sequences by database searching have been modified to address this challenge—in particular, addressing the decreased sensitivity of peptide matches due to increased false discovery rates in large databases [18], and challenges of protein identity inference due to sharing of proteins across multiple organisms in the database (e.g., the meta-protein concept in [22]).

Another significant challenge presented by metaproteomic informatics is that many disparate, specialized software tools must be used within each of the core areas required for successful data analysis and interpretation. For most researchers, these programs are difficult to access, master and operate. This reality offers a significant barrier for many researchers who could otherwise benefit from using metaproteomics approaches in their research.

Here we introduce new resources aimed at increasing access to advanced metaproteomic informatics tools and facilitating training in their use, thereby breaking down the barriers that hold back many researchers seeking to utilize metaproteomics in their work. The tools are housed in the Galaxy for proteomics (Galaxy-P) platform [23,24], which offers a user-friendly interface. Disparate software tools can be accessed and operated in an automated manner within a unified operating environment, which can be scaled to meet the demands of large-scale data analysis and informatics, as is often required in multi-omic approaches such as metaproteomics [24,25]. These resources were developed via a unique community-based effort, which leverages a consortium of leading experts from the metaproteomics research community, including a mixture of developers, data scientists and wet-bench researchers. These researchers participated in a contribution-fest (see z.umn.edu/mphack2016 for more information), wherein specific software was selected, deployed, tested and optimized within the Galaxy framework. In this manuscript, we describe not only the resources we have made available through this community-based effort, but also the process used to successfully achieve our goals. The accessible resources should help to increase wider adoption of metaproteomic informatics tools, as well as provide a framework for future collaborative efforts to make cutting-edge metaproteomic informatics tools available to the greater research community.

## 2. The Metaproteomics Gateway

### 2.1. Description of the Accessible Resources

Metaproteomics analysis of mass spectrometry data involves multiple core steps including database generation, MS/MS spectral matching to peptide sequences, taxonomic analysis and functional analysis. Below, we describe the general strategies and software currently available within these core areas, along with the process by which our consortium selected tools for deployment and dissemination via Galaxy-P. Since the main goal of this work, was to provide documentation to facilitate training and mastery of these software and workflows, we have provided step-by-step training instructions and related information in Supplement S (z.umn.edu/supps1). We have built a publicly accessible metaproteomics instance, or gateway (z.umn.edu/metaproteomicsgateway), for the purposes of providing access to documentation and other instructional materials, and an opportunity for hands-on training using example datasets and optimized metaproteomics workflows (See Table 1). Full instructions are provided at this site for registering in this gateway and gaining access to all materials.

### 2.2. The Playground: The Galaxy-P Platform

**Figure 2:**
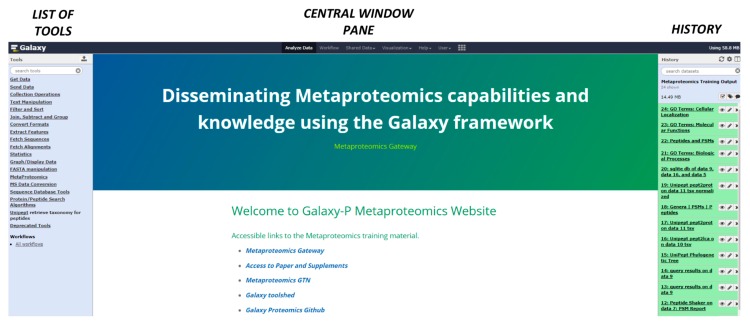
Galaxy interface and metaproteomics gateway. The Galaxy interface includes a tool menu, which consists of the list of available customized software within the instance in use. The central main viewing pane offers an area to view parameters for tools, edit workflows, and to visualize the results. The history menu maintains a real-time record of inputs and intermediate or final outputs from active software operations as the data is processed.

Galaxy-P is an extension of the open-source, Galaxy bioinformatics platform, which utilizes a web-based interface to access any instance, whether housed locally or remotely. The Galaxy interface includes a **Tool menu** (on the left of the screen—Figure 2), **Central main viewing pane** and the **History menu** (on the right side of the screen—Figure 2). 

### 2.3. The First Step: Protein Sequence Database Generation Using a Galaxy-Based Tool

The composition of the protein sequence database used to match MS/MS spectra to sequences has a profound effect on the depth and reliability of identified peptides and inferred proteins in metaproteomics [14]. The source of the sample, sample preparation methods utilized, and the focus of the specific study all play a role in determining the composition of the protein sequence database. The results are only as good as the sequence database used—for example if a peptide sequence present in the sample is not present in the database, neither the peptide, nor the protein it is associated with can be identified. Conversely, if the protein sequence database includes many proteins that are not actually contained in the sample being analyzed (e.g., a database containing all known bacterial proteins), the database size can be so large that it decreases the sensitivity for identifying peptides that are truly in the sample. Thus, generating optimized databases for metaproteomics is not trivial. Ideally, the database would be constructed based on the known taxonomic makeup of the sample being analyzed—which can be achieved by metagenomic analysis of the sample or by selecting publicly available taxonomic metagenomics databases, if these exist for the sample in question.

During the contribution fest, several options for protein sequence database generation were considered. We first looked at options already available within the Galaxy-P platform. One option was the use of publicly available taxonomic repositories specific to certain sample types or environments [26,27,28,29,30,31]. A tool in Galaxy-P (Protein Database Downloader) was already in place for automated generation of databases based on information available from repositories including the Human Microbiome Project, the Human Oral Microbiome database, and the EBI metagenomics resource. 

Another option already available within the Galaxy-P suite of tools is a tool for generating customized protein sequence databases from a list of genera thought to be in a sample. In some cases, a list of genera is available through previous published studies and can be useful in generating a protein sequence database [32,33]. In particular, 16S rRNA sequencing is used to assign operational taxonomic units (OTUs) in the form of species, genera or phyla. This can serve as a guide for generating a customized protein sequence database. Galaxy-P houses a tool to work through the UniProt Application Programming Interface (API) and extract protein sequences for all of the genera or phyla within a given list, generating a customized database for the metaproteomic analysis. 

Given these already existing tools, we decided to direct our efforts to deploying more cutting-edge tools for database generation, which follows recent trends in using metagenomics information to generate more accurate protein sequence databases tailored to the taxonomic make-up of any given sample [33,34,35,36,37]. In particular, whole metagenome sequencing offers increased taxonomic resolution over 16S rRNA sequencing, thus enabling more accurate taxonomic and functional categorization of identified sequences [38]. 

Targeting tools that leveraged emerging methods in whole metagenome sequencing, we considered two approaches. One is the recently described Omega (overlap-graph metagenome assembler), a software tool for assembly of shotgun metagenome data that can be used along with the Sipros algorithm for database generation and matching to MS/MS data [39]. The second was a novel method and software (called Sixgill) described by May et al. that uses a ‘metapeptide database’ derived from shotgun metagenomics sequencing [15]. The database generated using this method is optimized for MS/MS data, thereby providing a more rapid and accurate peptide to spectrum matching. In the original publication, the method was used on two ocean samples that had undergone whole genome metagenomics sequencing, and was shown to offer a significant increase in the number of identifications (presumably due to a more accurate and compact database) as compared to a metaproteome sequence database assembled using standard methods, as well as using the comprehensive sequence database from the NCBI repository.

**Figure 3:**
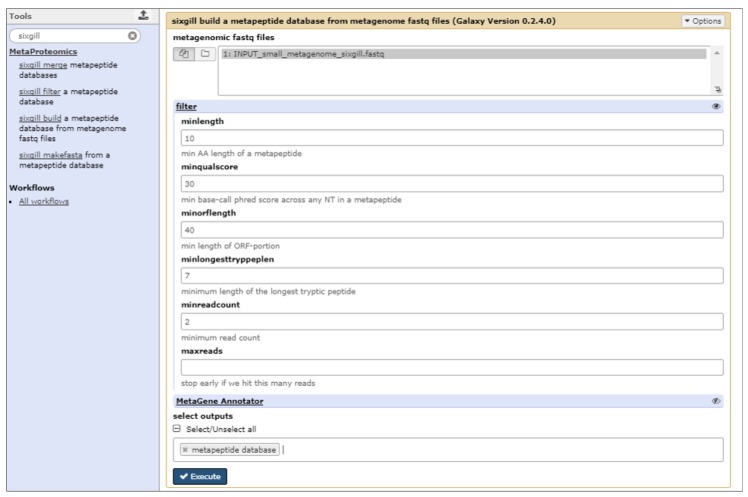
Sixgill tool within Galaxy. The Sixgill tool within Galaxy shows the build module, which uses a shotgun sequencing generated FASTQ file as an input, and generates a Tab-Separated Values (TSV) format file as an output. The filtering parameters aid in determining the quality and features of the output and are dependent on minimum length of the gene sequence, quality score, etc.

Given its demonstrated performance and optimized algorithm for utilizing large-scale, whole genome sequence data, we chose to implement the Sixgill software in Galaxy-P (Figure 3). We have provided step-by-step instructions for the use of Sixgill to create a metapeptide database, as well as the necessary input data, as described in Supplement S1. The deployed Sixgill tool provides a ‘build’ function, which generates a tab separated value (TSV) file containing the amino acid sequence of metapeptides along with other metrics. The Sixgill ‘makefasta’ function utilizes this information to generate a FASTA-formatted peptide database, which is compatible with database searching programs.

### 2.4. The Next Steps: Using a Galaxy Workflow

Galaxy also offers an option of generating a Galaxy ‘workflow’ which contains all the processing steps and software tool parameters for a particular analysis—except for the input or output data. Usually, workflows consist of multiple software tools, which are run in an automated, sequential manner, where outputs from one tool provide the input data for the next tool—ideally suited for multi-step analyses that are inherent to metaproteomic data analysis. Once built and optimized, workflows can be saved such that they become a main operational unit for analyzing different datasets in an efficient manner. Saved workflows can be also shared with other Galaxy users—thus promoting dissemination, reproducibility and collaboration.

**Figure 4:**
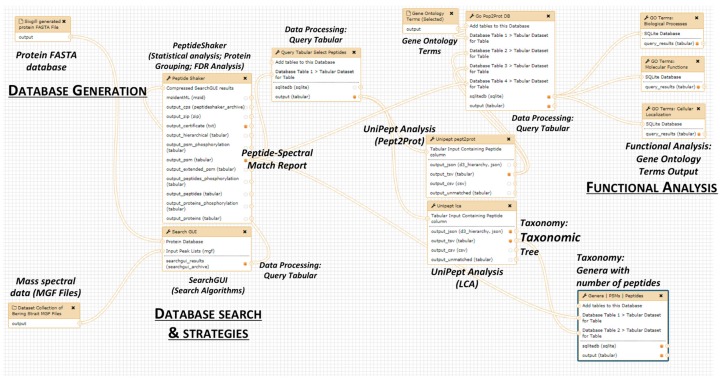
Edit view of Galaxy workflow for metaproteomics analysis. Representation of software tools used in a Galaxy metaproteomics workflow to identify bacterial peptides from the metaproteomic dataset. The first part of workflow includes database generation, followed by peak processing. The outputs from these sections are used for database search to generate a list of both bacterial peptide-spectral matches (PSMs). Later, bacterial PSMs were parsed out and subjected to Unipept analysis using Pept2Pro algorithm to generate outputs for functional analysis. Gene ontology categories such as biological processes, cellular localization and molecular function are generated. Additionally, bacterial PSMs were subjected to Unipept analysis using the lowest common ancestor algorithm to generate outputs for taxonomic analysis.

The remaining three steps comprising our metaproteomics informatics resource (spectral matching, taxonomy analysis and functional analysis) are encapsulated in a single workflow (Figure 4). The starting data inputs to this workflow are MS/MS data files (in the form of mascot generic files, MGFs) and the FASTA-formatted metapeptide sequence database generated in step 1 above. The second step (spectral matching) yields identified metapeptides that act as inputs for the third step (taxonomy analysis) and fourth step (functional analysis). For functional analysis, an additional input file with Gene Ontology (GO) terms is also required.

In our specific workflow built for training purposes, MGF files (from Bering Strait ocean samples) are searched against the metapeptide database (generated using Sixgill software on metagenomics data) as inputs. In order to save time, we have trimmed the MGF datasets and the Bering Strait metapeptide database from those provided in the manuscript by May et al. [15]. Users are recommended to refer to Supplement S1 for detailed instructions on how to use the workflow on the example dataset.

### 2.5. The Second Step: Spectral Matching

Sequence database searching algorithms that are able to match MS/MS spectra to peptide sequences contained in large databases (e.g., 10^6^ or more sequences) have also been developed specifically for metaproteomics applications [40,41]. Selecting from the available software for metaproteomic sequence database searching must balance the following factors: (a) ability to effectively use large databases while still sensitively matching spectra to peptide sequences; (b) speed of the core algorithm, along with scalability for execution on parallel computing infrastructure, enabling the processing of large datasets using large sequence databases in a reasonable timeframe; and (c) the ability to generate outputs with robust false discovery rate (FDR) estimations, that are also compatible with downstream processing steps for taxonomic and functional analysis. 

Multiple strategies have been suggested to increase the sensitivity of peptide identifications for the large sequence databases encountered in metaproteomics. This includes an iterative database searching workflow [42], a cascaded database search method [43] and a two-step method for searching large databases [44,45]. Muth et al. have recommended using a database sectioning approach, such that searches against subsets of a large database may increase the number of high confidence identifications [18]. The same group has proposed the use of de novo spectral matching in tandem with traditional sequence database-dependent methods [18], as well as the use of multiple database search algorithms, such as those offered by the SearchGUI tool [46], to increase the numbers of confident metapeptide identifications. 

For the workflow deployed in our informatics resource, we chose a relatively straightforward approach for spectral matching. We used the SearchGUI tool already deployed in Galaxy, utilizing X!Tandem as the sequence database search algorithm of choice. Although the Galaxy-deployed SearchGUI tool offers the use of multiple database search algorithms (e.g., MS-GF+, Myrimatch, OMSSA, Comet, Myrimatch, MS-Amanda and Novor), X!Tandem was determined to have a balance of speed and sensitivity that made it a good choice, especially for a training resource. The outputs from SearchGUI are further filtered and statistically analyzed using the companion PeptideShaker tool [47], which provides outputs compatible with downstream processing. Supplement S1 provides detailed instructions on the sequence database-searching step in this workflow, including a description of the small-scale input data we have provided for training purposes.

### 2.6. The Third Step: Taxonomic Classification

In metaproteomic studies, the identified microbial peptides can be used to determine the taxonomic composition of the sample. A number of options exist for taxonomic classification from the metapeptide data, some which were already deployed in Galaxy-P. The Unipept tool, deployed previously in Galaxy-P [24], maps sequences to annotated microbial organisms contained in the UniProt knowledgebase and subjects these to lowest common ancestor (LCA) analysis to provide a list of taxon identifications (at the level of kingdom, phylum, genus or species, if possible). The BLAST-P tool, also previously implemented in Galaxy-P [23], can match peptides to microbial proteins contained in the comprehensive NCBI non-redundant (nr) database, followed by taxonomic classification using MEGAN software [48] for metaproteomics data analysis [44].

During the metaproteomics contribution-fest, a number of new tools and extensions to new tools were considered for deployment in Galaxy. For example, taxonomy classification tools from the MetaProteomeAnalyzer [22] were considered, which process peptides identified via multiple database searching engines using information from the UniProt and National Center for Biotechnology Information (NCBI) repositories. Another tool under consideration was Prophane (https://mikrobiologie.uni-greifswald.de/en/resources/metaproteomics-data-analyses/prophane/), which uses the CLUSTAL W sequence alignment tool and other annotation tools to perform taxonomic classification.

**Figure 5:**
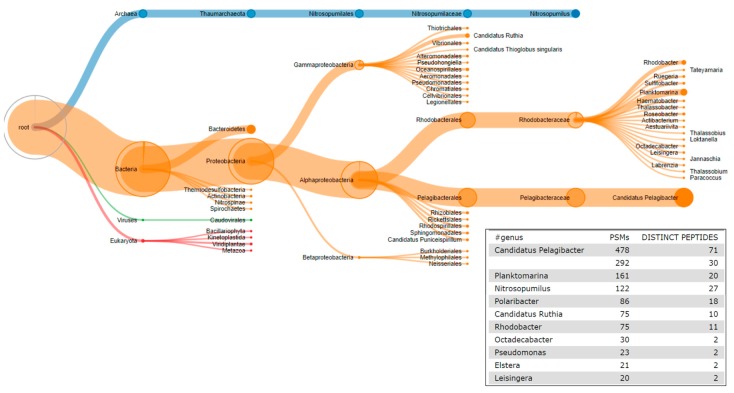
Taxonomy analysis using Unipept. Bacterial PSMs were subjected to Unipept analysis against UniProt database using lowest common ancestor algorithm to generate outputs for taxonomic analysis. These outputs include a Unipept Viewer which is an interactive visualization plugin that can be used to visualize taxonomic distribution of the ocean metaproteomic dataset. Unipept also generates a Comma-Separated Values (CSV) format file that lists the peptide assignments to taxa. That file then can be parsed to generate a tabular output (lower right).

Ultimately, the work stemming from the contribution fest focused on extending the functionality in Galaxy-P of the Unipept tool [19,49,50]. As mentioned above, Unipept was already deployed in Galaxy-P, providing textual outputs of taxonomic classes (Figure 5). We extended this function, adding the capability of visualizing taxonomic groups by packaging recently added visualization capabilities of Unipept into the Galaxy-based tool (Figure 5). With this functionality, the outputs from the metaproteomics workflow run in Galaxy-P now offers the user the option of launching a visualization window of the taxonomic results. (Figure 4). Details about this functionality within the workflow are provided in Supplement S1.

### 2.7. The Fourth Step: Functional Analysis

Metaproteomics has a distinct advantage in determining the functional signature associated with a microbial community under a specific condition based on identification of the proteins that are actually being expressed [44]. However, characterizing the functional state from a collection of expressed proteins is not trivial. Functional annotation based on a protein profile requires several components: a controlled vocabulary (or ontology) that represents protein function, databases containing annotations of known proteins or protein families with terms from these vocabularies, and alignment tools that map functional annotations within data repositories to the experimentally identified peptides or proteins. Many ontologies exist and often focus on different aspects of function: the Gene Ontology [51] and Kyoto Encyclopedia of Genes and Genomes (KEGG) pathways [52] are two of the most prominent. A number of databases use diverse methodologies to assign function to proteins and or its groups—these include the InterPro [53], and “evolutionary genealogy of genes: Non-supervised Orthologous Groups” (eggnog) [54] databases. Finally, tools to map functional annotations from these databases to experimentally identified proteins are often database-specific, such as the eggNOG-mapper [55] and InterProScan [56]. In addition, MEGAN6 can be used to carry out InterPro2GO, KEGG, SEED and EggNOG analysis to determine the distribution of functions amongst expressed proteins in the microbiome [48]. 

Beyond mapping functional annotations to identified proteins, visualization of the collective functional categories is the next desirable step. Here, various options are available—with potential for deployment in the Galaxy platform. For single GO terms of interest, the QuickGO browser [57] enables the user to view the full term definition, as well as to browse closely related terms. For large lists of GO terms, the ‘reduce and visualize Gene Ontology’ (REVIGO) tool [58] allows the reduction of GO terms to a representative subset and several visualizations of the resulting smaller list. Moreover, the Prophane suite of tools can also be used to determine the distribution of functions in a microbiome sample and visualize them. MetaProteomeAnalyzer provides enzyme and pathway display options where proteins grouped by UniProt ontologies (e.g., biological process or molecular function), EC (Enzyme Commission) numbers and KEGG pathways can be visualized [22].

**Figure 6:**
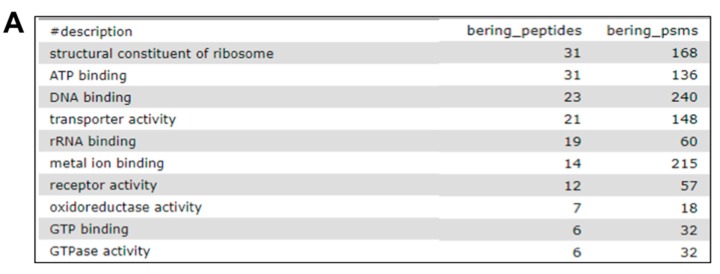

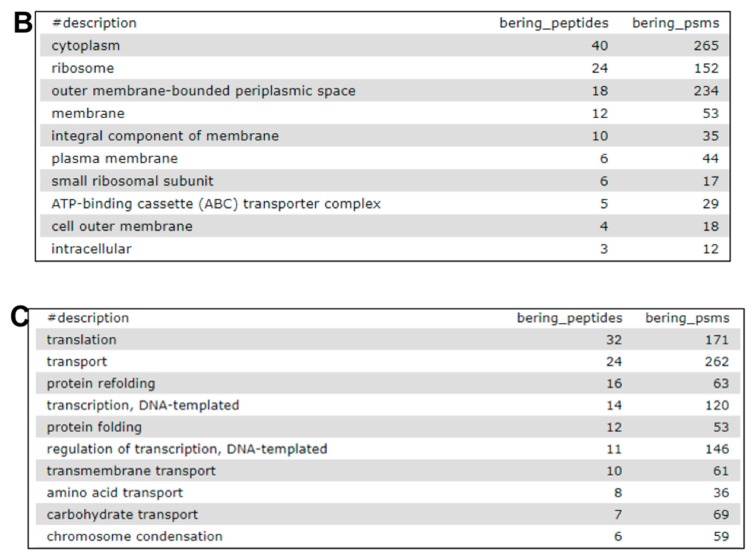
Functional analysis using Unipept and GO (Gene Ontology) terms. Bacterial PSMs were subjected to Unipept analysis against Pept2Pro algorithm to generate outputs for functional analysis. Using PSM report, gene ontology mapping files and Unipept outputs, the query tabular file generates tabular outputs for gene ontology categories. The generated tabular outputs for molecular function (**A**), cellular localization; (**B**) and biological processes; (**C**) and also enlist the number of associated peptides and PSMs with each gene ontology category.

Although all of these options have great potential for functional annotation and visualization, our community-based efforts focused on utilizing the Galaxy-deployed Unipept tool and its Pept2Prot option, which maps identified peptide sequences to proteins. The proteins are then mapped to GO terms for molecular function, biological processes and cellular localization, followed by using the GO term mapping information (Figure 6). The grouping into functional categories was performed using a Galaxy tool, query tabular tailored to automate extraction and grouping of tabular data results. These results are presented as a tabular output for further downstream analysis, such as visualization software. Details are provided in Supplement S1 about the tools involved in this functional annotation step, along with instructions.

### 2.8. Links to Accessible Resources for Training

The main goal of our contribution fest was to provide an instrument for researchers to access and learn the operation of cutting-edge metaproteomics tools. We have provided several means for researchers to access and train in the operation of these tools (See Table 1). We have established a Metaproteomics Gateway, composed of a publicly accessible Galaxy instance containing the tools, workflows and example data described in this manuscript. Supplement S1 provides a detailed description for the use of this gateway. We have also provided our documentation and training instructions within the Galaxy Training Network repository (http://galaxyproject.github.io/training-material/), a central resource for providing documentation on Galaxy-based tools and platforms. Our tools and workflows have also been made openly available through the Galaxy Tool Shed and on GitHub. We hope that the available resources that also include an introductory video will encourage researchers to incorporate metaproteomics studies into their current expertise of research.

In conclusion, we have described accessible resources aimed at training researchers in the use of advanced metaproteomic informatics tools, with the intent of increasing the adoption of metaproteomics by the wider research community. These tools have been made available through a unique, community-based process, which has leveraged a community of metaproteomic informatics experts, as well as the powerful Galaxy platform. We would like to emphasize that the use of Galaxy was highly enabling for this work, as it provides a unified environment for operating many disparate tools required in metaproteomics, as well as a platform that can be used to promote training and usage by the larger community.

Several other points are worth noting from the work we have described here. It is evident that, for each of the core steps described in the metaproteomic data analysis pipeline, there are many valuable software tools that already exist. During our contribution fest, our consortium of researchers were only able to deploy, test and optimize a select few of these tools. Work is ongoing on implementing additional tools. In the future, we anticipate increased need for visualization, quantitation and statistical tools in metaproteomics research, which will aid in biological interpretation. It is our hope that this manuscript serves as an invitation to others to join our collaborative community and help to make additional high-value tools for metaproteomics available. Again, our usage of the open source Galaxy-P platform for deployment and dissemination provides a playground for developers to come ‘play’ in, and collaborate with other like-minded researchers from around the world. We also hope that the ‘shareable’ workflows developed will facilitate the undertaking of global scale research projects.

It is our hope that this manuscript will help establish a framework for continued, community-based efforts at making cutting-edge metaproteomics tools available to others, along with the necessary documentation and hands-on training resources to educate researchers in their use. Ultimately, we hope this approach will yield great dividends in increasing the adoption of metaproteomic approaches by more researchers, which will help catalyze a better understanding of the molecular characteristics of dynamic microbial communities and microbiome.

## Figures and Tables

**Table 1:** Links to the resources for metaproteomics training.

**Metaproteomics Gateway**	*z.umn.edu/metaproteomicsgateway*
**Galaxy Training Network**	*http://galaxyproject.github.io/training-material/topics/proteomics/tutorials/metaproteomics/tutorial.html*
**Documentation**	*Supplement S1*
**Introductory video**	*z.umn.edu/mpvideo2018*
**Galaxy toolshed**	*https://toolshed.g2.bx.psu.edu/*
**GitHub**	*https://github.com/galaxyproteomics*

## Supplementary Materials

The following are available online at http://www.mdpi.com/2227-7382/6/1/7/s1.

## References

[1] Knight R., Callewaert C., Marotz C., Hyde E.R., Debelius J.W., McDonald D., Sogin M.L. (2017). The Microbiome and Human Biology. Annu. Rev. Genom. Hum. Genet..

[2] Foo J.L., Ling H., Lee Y.S., Chang M.W. (2017). Microbiome engineering: Current applications and its future. Biotechnol. J..

[3] Arnold J.W., Roach J., Azcarate-Peril M.A. (2016). Emerging Technologies for Gut Microbiome Research. Trends Microbiol..

[4] Siegwald L., Touzet H., Lemoine Y., Hot D., Audebert C., Caboche S. (2017). Assessment of Common and Emerging Bioinformatics Pipelines for Targeted Metagenomics. PLoS ONE.

[5] Maier T.V., Lucio M., Lee L.H., VerBerkmoes N.C., Brislawn C.J., Bernhardt J., Lamendella R., McDermott J.E., Bergeron N., Heinzmann S.S. (2017). Impact of Dietary Resistant Starch on the Human Gut Microbiome, Metaproteome, and Metabolome. mBio.

[6] Heintz-Buschart A., May P., Laczny C.C., Lebrun L.A., Bellora C., Krishna A., Wampach L., Schneider J.G., Hogan A., de Beaufort C. (2016). Integrated multi-omics of the human gut microbiome in a case study of familial type 1 diabetes. Nat. Microbiol..

[7] Wilmes P., Bond P.L. (2006). Metaproteomics: Studying functional gene expression in microbial ecosystems. Trends Microbiol..

[8] Heintz-Buschart A., Wilmes P. (2017). Human Gut Microbiome: Function Matters. Trends Microbiol..

[9] Wilmes P., Heintz-Buschart A., Bond P.L. (2015). A decade of metaproteomics: Where we stand and what the future holds. Proteomics.

[10] Tanca A., Abbondio M., Palomba A., Fraumene C., Manghina V., Cucca F., Fiorillo E., Uzzau S. (2017). Potential and active functions in the gut microbiota of a healthy human cohort. Microbiome.

[11] Human Microbiome Project Consortium (2012). A framework for human microbiome research. Nature.

[12] Tanca A., Palomba A., Fraumene C., Pagnozzi D., Manghina V., Deligios M., Muth T., Rapp E., Martens L., Addis M.F. (2016). The impact of sequence database choice on metaproteomic results in gut microbiota studies. Microbiome.

[13] Tanca A., Palomba A., Deligios M., Cubeddu T., Fraumene C., Biosa G., Pagnozzi D., Addis M.F., Uzzau S. (2013). Evaluating the impact of different sequence databases on metaproteome analysis: Insights from a lab-assembled microbial mixture. PLoS ONE.

[14] Timmins-Schiffman E., May D.H., Mikan M., Riffle M., Frazar C., Harvey H.R., Noble W.S., Nunn B.L. (2017). Critical decisions in metaproteomics: Achieving high confidence protein annotations in a sea of unknowns. ISME J..

[15] May D.H., Timmins-Schiffman E., Mikan M.P., Harvey H.R., Borenstein E., Nunn B.L., Noble W.S. (2016). An Alignment-Free “Metapeptide” Strategy for Metaproteomic Characterization of Microbiome Samples Using Shotgun Metagenomic Sequencing. J. Proteome Res..

[16] Tang H., Li S., Ye Y. (2016). A Graph-Centric Approach for Metagenome-Guided Peptide and Protein Identification in Metaproteomics. PLoS Comput. Biol..

[17] Muth T., Renard B.Y., Martens L. (2016). Metaproteomic data analysis at a glance: Advances in computational microbial community proteomics. Expert Rev. Proteom..

[18] Muth T., Kolmeder C.A., Salojärvi J., Keskitalo S., Varjosalo M., Verdam F.J., Rensen S.S., Reichl U., de Vos W.M., Rapp E. (2015). Navigating through metaproteomics data: A logbook of database searching. Proteomics.

[19] Mesuere B., Debyser G., Aerts M., Devreese B., Vandamme P., Dawyndt P. (2015). The Unipept metaproteomics analysis pipeline. Proteomics.

[20] Xiong W., Brown C.T., Morowitz M.J., Banfield J.F., Hettich R.L. (2017). Genome-resolved metaproteomic characterization of preterm infant gut microbiota development reveals species-specific metabolic shifts and variabilities during early life. Microbiome.

[21] Huson D.H., Beier S., Flade I., Górska A., El-Hadidi M., Mitra S., Ruscheweyh H.J., Tappu R. (2016). MEGAN Community Edition—Interactive Exploration and Analysis of Large-Scale Microbiome Sequencing Data. PLoS Comput. Biol..

[22] Muth T., Behne A., Heyer R., Kohrs F., Benndorf D., Hoffmann M., Lehtevä M., Reichl U., Martens L., Rapp E. (2015). The MetaProteomeAnalyzer: A powerful open-source software suite for metaproteomics data analysis and interpretation. J. Proteome Res..

[23] Jagtap P.D., Johnson J.E., Onsongo G., Sadler F.W., Murray K., Wang Y., Shenykman G.M., Bandhakavi S., Smith L.M., Griffin T.J. (2014). Flexible and accessible workflows for improved proteogenomic analysis using the Galaxy framework. J. Proteome Res..

[24] Jagtap P.D., Blakely A., Murray K., Stewart S., Kooren J., Johnson J.E., Rhodus N.L., Rudney J., Griffin T.J. (2015). Metaproteomic analysis using the Galaxy framework. Proteomics.

[25] Afgan E., Baker D., van den Beek M., Blankenberg D., Bouvier D., Čech M., Chilton J., Clements D., Coraor N., Eberhard C. (2016). The Galaxy platform for accessible, reproducible and collaborative biomedical analyses: 2016 update. Nucleic Acids Res..

[26] Wilmes P., Bond P.L. (2004). The application of two-dimensional polyacrylamide gel electrophoresis and downstream analyses to a mixed community of prokaryotic microorganisms. Environ. Microbiol..

[27] Klaassens E.S., de Vos W.M., Vaughan E.E. (2007). Metaproteomics approach to study the functionality of the microbiota in the human infant gastrointestinal tract. Appl. Environ. Microbiol..

[28] Rudney J.D., Xie H., Rhodus N.L., Ondrey F.G., Griffin T.J. (2010). A metaproteomic analysis of the human salivary microbiota by three-dimensional peptide fractionation and tandem mass spectrometry. Mol. Oral Microbiol..

[29] Haange S.B., Oberbach A., Schlichting N., Hugenholtz F., Smidt H., von Bergen M., Till H., Seifert J. (2012). Metaproteome analysis and molecular genetics of rat intestinal microbiota reveals section and localization resolved species distribution and enzymatic functionalities. J. Proteome Res..

[30] Jagtap P., McGowan T., Bandhakavi S., Tu Z.J., Seymour S., Griffin T.J., Rudney J.D. (2012). Deep metaproteomic analysis of human salivary supernatant. Proteomics.

[31] Bastida F., Hernández T., García C. (2014). Metaproteomics of soils from semiarid environment: Functional and phylogenetic information obtained with different protein extraction methods. J. Proteom..

[32] Wu J., Zhu J., Yin H., Liu X., An M., Pudlo N.A., Martens E.C., Chen G.Y., Lubman D.M. (2016). Development of an Integrated Pipeline for Profiling Microbial Proteins from Mouse Fecal Samples by LC-MS/MS. J. Proteome Res..

[33] Kohrs F., Wolter S., Benndorf D., Heyer R., Hoffmann M., Rapp E., Bremges A., Sczyrba A., Schlüter A., Reichl U. (2015). Fractionation of biogas plant sludge material improves metaproteomic characterization to investigate metabolic activity of microbial communities. Proteomics.

[34] Bao Z., Okubo T., Kubota K., Kasahara Y., Tsurumaru H., Anda M., Ikeda S., Minamisawa K. (2014). Metaproteomic identification of diazotrophic methanotrophs and their localization in root tissues of field-grown rice plants. Appl. Environ. Microbiol..

[35] Colatriano D., Ramachandran A., Yergeau E., Maranger R., Gélinas Y., Walsh D.A. (2015). Metaproteomics of aquatic microbial communities in a deep and stratified estuary. Proteomics.

[36] Young J.C., Pan C., Adams R.M., Brooks B., Banfield J.F., Morowitz M.J., Hettich R.L. (2015). Metaproteomics reveals functional shifts in microbial and human proteins during a preterm infant gut colonization case. Proteomics.

[37] Mattarozzi M., Manfredi M., Montanini B., Gosetti F., Sanangelantoni A.M., Marengo E., Careri M., Visioli G. (2017). A metaproteomic approach dissecting major bacterial functions in the rhizosphere of plants living in serpentine soil. Anal. Bioanal. Chem..

[38] Jovel J., Patterson J., Wang W., Hotte N., O’Keefe S., Mitchel T., Perry T., Kao D., Mason A.L., Madsen K.L. (2016). Characterization of the Gut Microbiome Using 16S or Shotgun Metagenomics. Front. Microbiol..

[39] Haider B., Ahn T.H., Bushnell B., Chai J., Copeland A., Pan C. (2014). Omega: An overlap-graph de novo assembler for metagenomics. Bioinformatics.

[40] Chatterjee S., Stupp G.S., Park S.K., Ducom J.C., Yates J.R., Su A.I., Wolan D.W. (2016). A comprehensive and scalable database search system for metaproteomics. BMC Genom..

[41] Guo X., Li Z., Yao Q., Mueller R.S., Eng J.K., Tabb D.L., Hervey W.J., Pan C. (2017). Sipros Ensemble Improves Database Searching and Filtering for Complex Metaproteomics. Bioinformatics.

[42] Rooijers K., Kolmeder C., Juste C., Doré J., de Been M., Boeren S., Galan P., Beauvallet C., de Vos W.M., Schaap P.J. (2011). An iterative workflow for mining the human intestinal metaproteome. BMC Genom..

[43] Kertesz-Farkas A., Keich U., Noble W.S. (2015). Tandem Mass Spectrum Identification via Cascaded Search. J. Proteome Res..

[44] Rudney J.D., Jagtap P.D., Reilly C.S., Chen R., Markowski T.W., Higgins L., Johnson J.E., Griffin T.J. (2015). Protein relative abundance patterns associated with sucrose-induced dysbiosis are conserved across taxonomically diverse oral microcosm biofilm models of dental caries. Microbiome.

[45] Jagtap P., Goslinga J., Kooren J.A., McGowan T., Wroblewski M.S., Seymour S.L., Griffin T.J. (2013). A two-step database search method improves sensitivity in peptide sequence matches for metaproteomics and proteogenomics studies. Proteomics.

[46] Vaudel M., Barsnes H., Berven F.S., Sickmann A., Martens L. (2011). SearchGUI: An open-source graphical user interface for simultaneous OMSSA and X!Tandem searches. Proteomics.

[47] Vaudel M., Burkhart J.M., Zahedi R.P., Oveland E., Berven F.S., Sickmann A., Martens L., Barsnes H. (2015). PeptideShaker enables reanalysis of MS-derived proteomics data sets. Nat. Biotechnol..

[48] Huson D.H., Weber N. (2013). Microbial community analysis using MEGAN. Methods Enzymol..

[49] Mesuere B., Van der Jeugt F., Willems T., Naessens T., Devreese B., Martens L., Dawyndt P. (2017). High-throughput metaproteomics data analysis with Unipept: A tutorial. J. Proteom..

[50] Mesuere B., Willems T., Van der Jeugt F., Devreese B., Vandamme P., Dawyndt P. (2016). Unipept web services for metaproteomics analysis. Bioinformatics.

[51] Gene Ontology Consortium (2012). The Gene Ontology: Enhancements for 2011. Nucleic Acids Res..

[52] Kanehisa M., Furumichi M., Tanabe M., Sato Y., Morishima K. (2017). KEGG: New perspectives on genomes, pathways, diseases and drugs. Nucleic Acids Res..

[53] Hunter S., Apweiler R., Attwood T.K., Bairoch A., Bateman A., Binns D., Bork P., Das U., Daugherty L., Duquenne L. (2009). InterPro: The integrative protein signature database. Nucleic Acids Res..

[54] Huerta-Cepas J., Szklarczyk D., Forslund K., Cook H., Heller D., Walter M.C., Rattei T., Mende D.R., Sunagawa S., Kuhn M. (2016). eggNOG 4.5: A hierarchical orthology framework with improved functional annotations for eukaryotic, prokaryotic and viral sequences. Nucleic Acids Res..

[55] Huerta-Cepas J., Forslund K., Coelho L.P., Szklarczyk D., Jensen L.J., von Mering C., Bork P. (2017). Fast Genome-Wide Functional Annotation through Orthology Assignment by eggNOG-Mapper. Mol. Biol. Evolut..

[56] Jones P., Binns D., Chang H.Y., Fraser M., Li W., McAnulla C., McWilliam H., Maslen J., Mitchell A., Nuka G. (2014). InterProScan 5: Genome-scale protein function classification. Bioinformatics.

[57] Binns D., Dimmer E., Huntley R., Barrell D., O’Donovan C., Apweiler R. (2009). QuickGO: A web-based tool for Gene Ontology searching. Bioinformatics.

[58] Supek F., Bošnjak M., Škunca N., Šmuc T. (2011). REVIGO summarizes and visualizes long lists of gene ontology terms. PLoS ONE.

