# Supplementary material for: Disseminating Metaproteomic Informatics Capabilities and Knowledge Using the Galaxy-P Framework
Source: Proteomes. 2018 Jan 31;6(1):7. doi: 10.3390/proteomes6010007 (PMC5874766; doi:10.3390/proteomes6010007)
Supplement: Supplementary file 1 [file proteomes-06-00007-s001.pdf]

<u>SUPPLEMENTARY DOCUMENTATION S1</u>

The Galaxy Instance used for our metaproteomics gateway can be accessed by using a web-based user interface accessed by the URL "z.umn.edu/metaproteomicsgateway". The Tool Pane (left side column) contains a list of available software tools in the Galaxy instance. The central portion of the interface is called the Main Viewing Pane, where the users can set operating parameters for the tools, edit and view workflows comprised of multiple tools, and also view results for and from data analyses. The right-side column of the interface is the History Pane, which shows the active History.

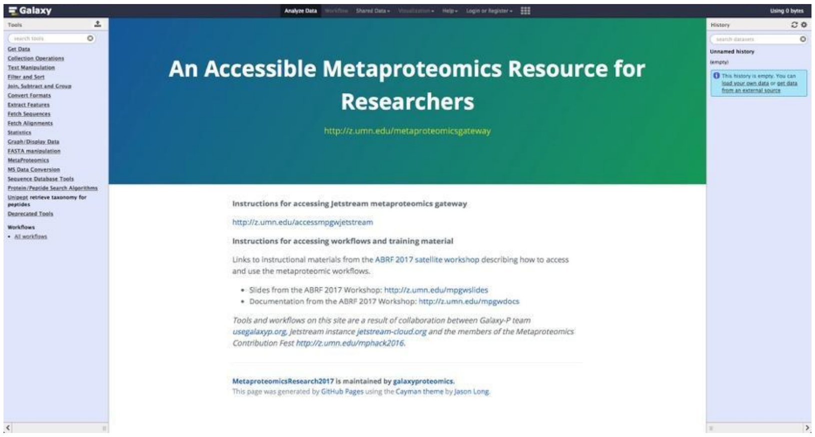

Firstly, in order to use the Galaxy instance, register as a user and create login/password credentials.

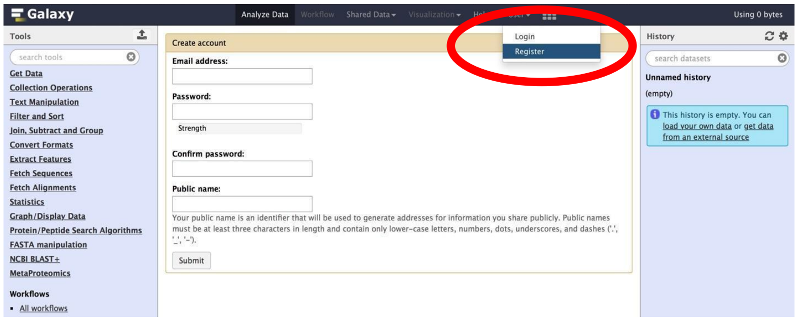

Using a Galaxy tool: Database generation

The first step in the analysis is to import the required input data files, which users can download from the Shared Data Libraries. Once imported, these data files will appear on the History pane. [click on Shared Data tab, and then click on "Data Libraries".

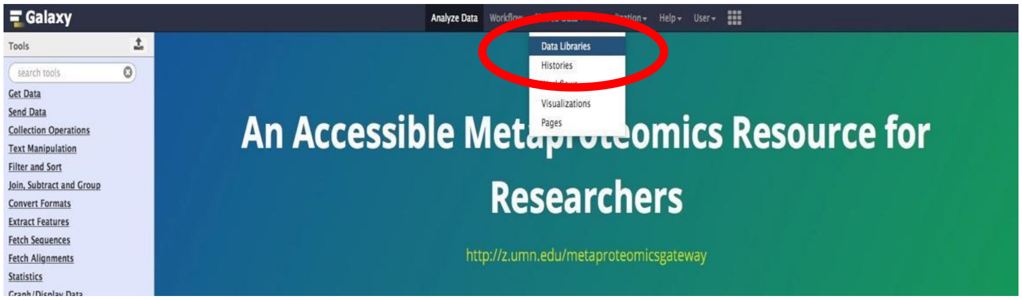

In the list of shared data, click on “Metaproteomics Training”. Click “Metagenome_sixgill.fastq”, select the file, and click on “Import to History”. This folder contains one file in the fastq format, which consists of the biological sequence and its corresponding quality scores data.

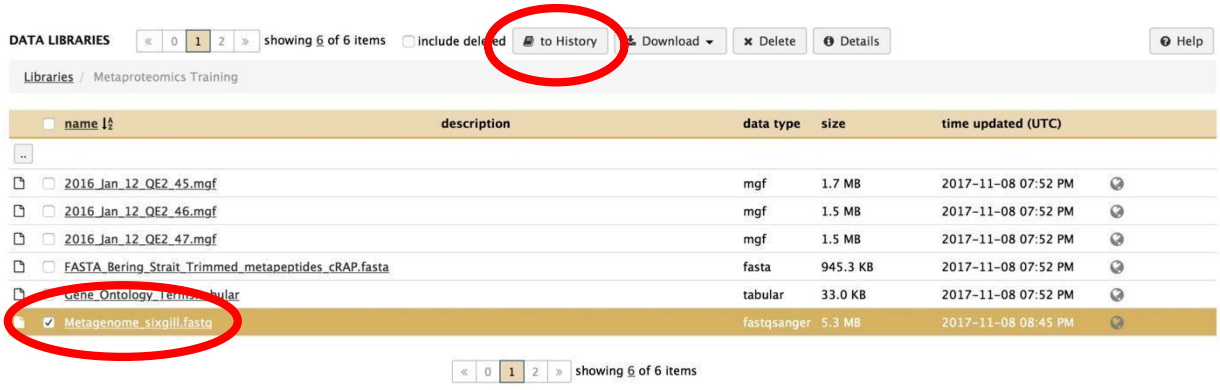

Sixgill (Six-frame Genome-Inferred Libraries for LC-MS/MS) is a tool for using shotgun metagenomics sequencing reads to construct databases of '**metapeptides**': short protein fragments for database search of LC-MS/MS metaproteomics data. The main Sixgill command is **sixgill_build** (http://noble.gs.washington.edu/proj/metapeptide/), which builds a metapeptide [illegible]

Once the file is imported to your history, click on ““Shared Data -> Workflows” and then on “Metagenomics to Metaproteomics for training”. Click on import workflow.

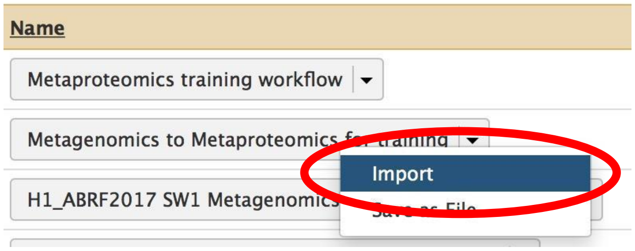

The workflow will be imported to your personal workflow library. Click on the “Workflow” tab and select the imported workflow. A drop-down menu appears, click on “Run”. The workflow will appear on the main viewing pane. Click on “Run workflow” which will select the input files and build a FASTA database.

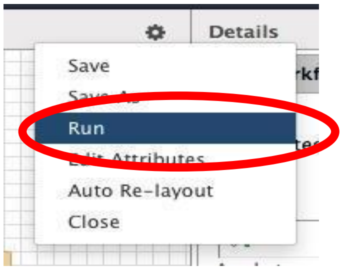

This is one way to perform Database generation. You could also use a publicly available database or use the Protein Database Downloader tool within Galaxy to download protein FASTA databases from single organism as well as metaproteomic databases.

The **Protein Database Downloader** tool helps in downloading a FASTA file of specified protein sequences for comparison with experimental MS/MS data in search algorithm. The Protein Database downloader can download sequences from Uniprot, cRAP, EBI Metagenomics, HOMD (Human Oral Microbiome Database) and Human Microbiome Project body sites (airways, blood, gastro-intestinal tract, oral, skin and urogenital tract). You can also download it through Custom-URL.

Using a Galaxy Workflow:

Now that the protein database ready, we will use the metaproteomics workflow on the MGF file inputs for database searching, taxonomy analysis and functional analysis. Firstly, the input files **Mascot generated format** (MGF) files need to be imported. Please note, that these are trimmed MGF files from Bering Strait dataset along with a trimmed Sixgill-generated metapeptide FASTA file (*J. Proteome Res.*, 2016, *15* (8), pp 2697–2705). For functional analysis, we will also need a Gene Ontology mapping file (http://geneontology.org/ontology/go-basic.obo).

To obtain these files, click on “Shared Data” and select “Data Libraries”. Click on “Metaproteomics training”. Select all the files in the folder (excluding the Metagenome Sixgill.fastq) and import it to history. Name the history as ‘Metaproteomics Training Output’. Once imported your history should have 3 MGF files, 1 Gene Ontology File and 1 metapeptide FASTA database. In order to prepare all the MGF fractions for database search (and subsequent steps) so that it generates a single output, we will need to create a Dataset collection. Select all the MGF files in your input history and create a Dataset collection.

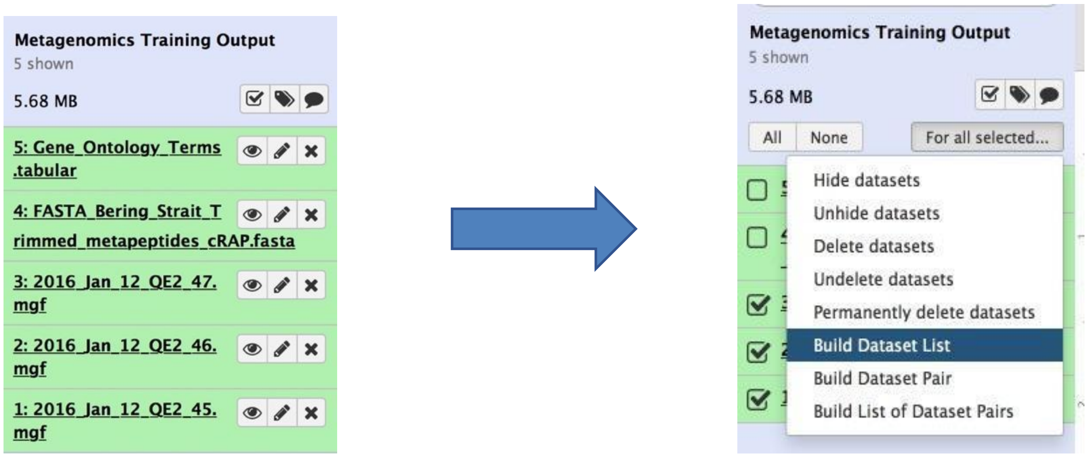

The Dataset collection can be labeled as “BeringStrait MGFs”. By creating a dataset collection, all the MGF files can be searched together to generate a single search output.

Now that you have inputs for running a workflow, we can import the Metaproteomics training workflow. For this, select “Shared Data -> Workflows” and click on “Metaproteomics training workflow” to import the workflow.

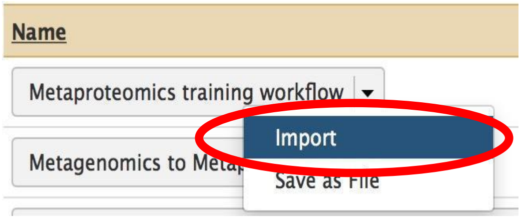

To run the workflow, go to the **“Workflow” tab** and select the recently imported workflow. When you run the workflow, it will appear on your Main viewing pane. Ensure that you see appropriately labeled input files in the boxes and click on **Run**.

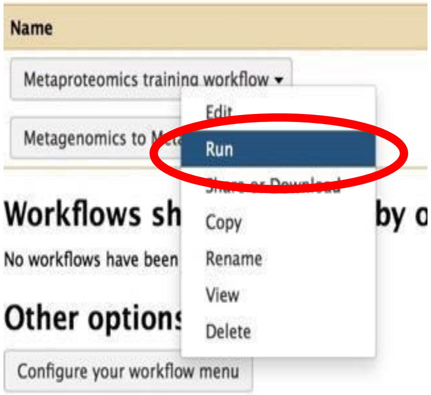

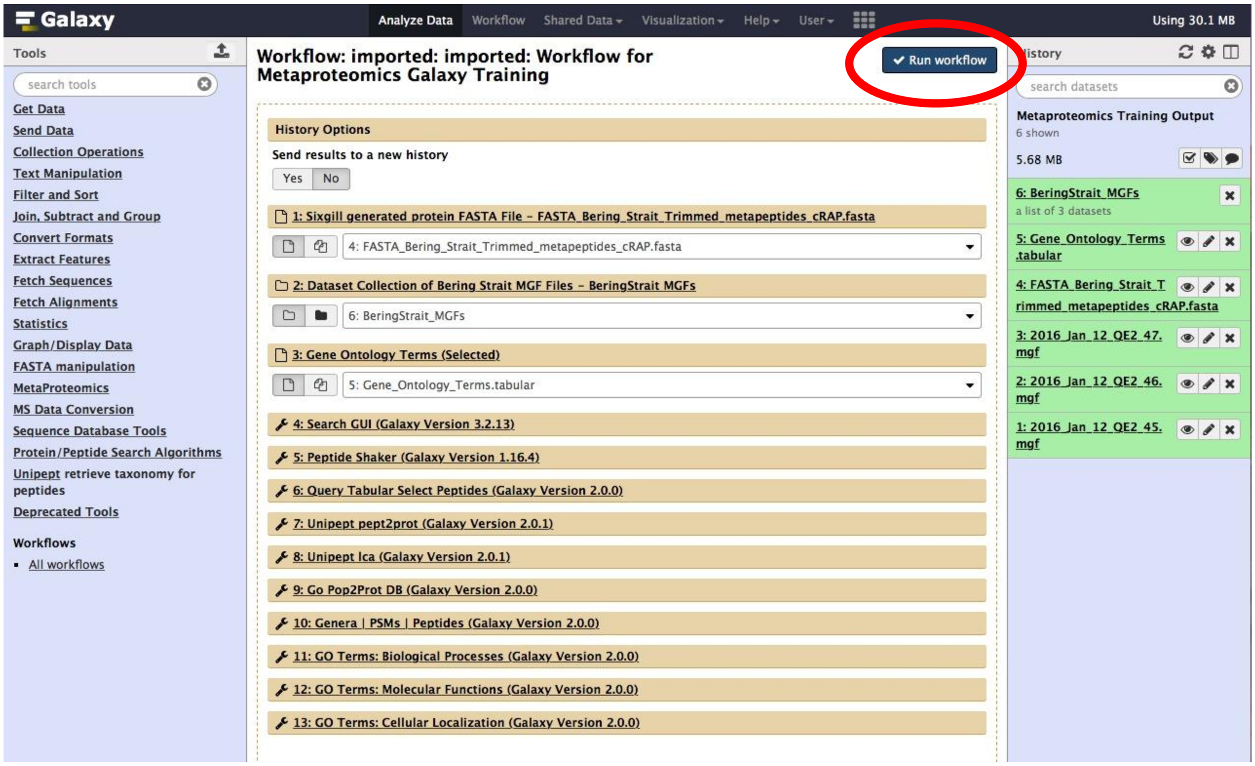

Once you click **Run Workflow**, all the tools mentioned in the workflow will queue up in the history panel. These tools will be grey while in queue, yellow while running and green in color once the task has finished. Once completed, the history pane on right would look like the one shown below:

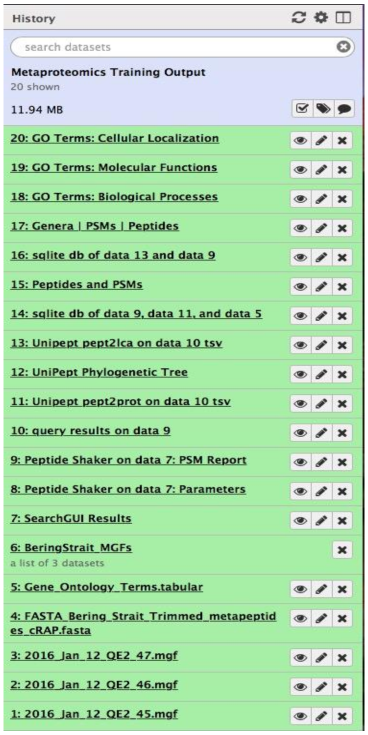

Workflow component: Spectral Matching

In the generated History, Steps **7-9** are related to database searching, where we use SearchGUI and PeptideShaker to search the MGF files against the Metapeptide Database.

SearchGUI (Proteomics, 2011, 11:996-999), bundles several open-source and freely available sequence database searching programs, facilitating analysis of MS/MS data using more than one algorithm and increasing confidence in results. SearchGUI has been deployed in Galaxy. Here we will use it to match MS/MS spectra to sequences in our FASTA database.

PeptideShaker (Nature Biotechnol. 2015, 33(1):22–24), runs multiple search engines (X! Tandem, OMSSA, MS-GF+ and others) on any number of MGF peaklists using the SearchGUI application and combines the results.

SearchGUI performs protein identification using various search engine, in this workflow for simplicity, we will only use one database search engine i.e. X!Tandem.

Parameters used for SearchGUI:

Protein digestion parameters: Trypsin, with 2 maximum missed cleavages
The precursor ion tolerance is 10 ppm, with fragment tolerance of 0.02 Da
Minimum/maximum charge of ions: 2/6
Fragment ions searched: b and y
Fixed protein modification: Carbamidomethylation of C
Variable protein modification: Oxidation of M

PeptideShaker processes the output file from SearchGUI. It infers proteins from matched peptide sequences and applies statistical metrics to assign confidence to identified peptides and proteins. Within this workflow, the "Default" options are selected, with relevant parameters as follows:

Parameters used for PeptideShaker:

The maximum FDR value (%) at protein level is 1.0, peptide level is 1.0 and PSM level is 1.0
Minimum and maximum peptide length are 6 and 30 respectively
Maximum precursor error is 10.0 ppm
Outputs selected: PSM report (tabular) and Certificate of Analysis (text).

For this workflow, the PSM report was selected along with the Certificate of Analysis. The History contains the PSM report generated for our workflow.

Workflow component: Data Processing

Step **10** utilizes the Query Tabular tool that was used to select those distinct PSMs that had a confidence of more than 95%.

Parameters for Query Tabular:

```
SELECT distinct sequence
FROM psm
WHERE confidence >= 95
ORDER BY sequence
```

The tabular output that contains distinct microbial peptides that is then subjected to UniPept Analysis (*Proteomics* 2015, **15**, 1437–1442).

Unipept (http://unipept.ugent.be/) is an open-source web application developed at Ghent University that is designed for metaproteomics data analysis with a focus on interactive taxonomic data visualizations. Unipept is powered by an index containing all Uniprot Entries, NCBI taxonomy and custom lowest common ancestor (LCA). This helps in performing bio-diversity analysis of large and complex metaproteome samples. It's available in API and command line interface. Unipept also has tools for selecting unique peptides for targeted proteomics for comparing genomes based on peptide similarity.

In this workflow, Unipept (Steps **11-13**) is used for both taxonomic and functional analysis. Detected peptides were given taxonomic assignments by Unipept version 2.0.1. For all tryptic peptides with no missed cleavages present in UniProtKB, Unipept assigns a lowest common ancestor (LCA) taxon from the NCBI Taxonomy Database, the most- granular taxon common to all organisms containing the peptide. For peptides with missed tryptic cleavages, Unipept calculates an LCA based on the LCAs associated with all completely cleaved peptide sequences contained in the peptide.

Workflow component: Taxonomy analysis

Taxonomy analysis (Step **12-13**) uses the Unipept pept2lca function to generate the taxonomic lowest common ancestor for each peptide. Unipept analysis using "pept2lca" function generates two outputs:
a. The JavaScript Object Notation (JSON) output, which will be used for visualization.
b. The tabular (.tsv) output

***Parameter used for Unipept Taxonomy Analysis**:*

<u>Unipept application: pept2lca: lowest common ancestor</u>

Equate isoleucine and leucine: YES
(isoleucine (I) and leucine (L) are equated when matching tryptic peptides to UniProt records)
retrieve extra information: NO
(Return the complete lineage of the taxonomic lowest common ancestor, and include ID fields.)
names: YES
return the names in complete taxonomic lineage

The JSON file (**Step 12**) opens a Unipept tree viewer in which user can interactively explore the taxonomy tree represented in our sample.

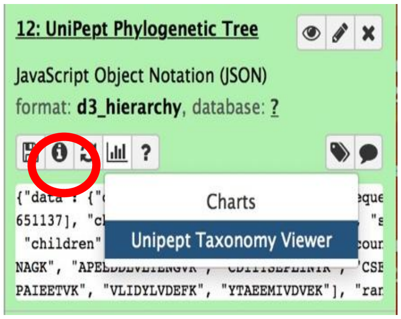

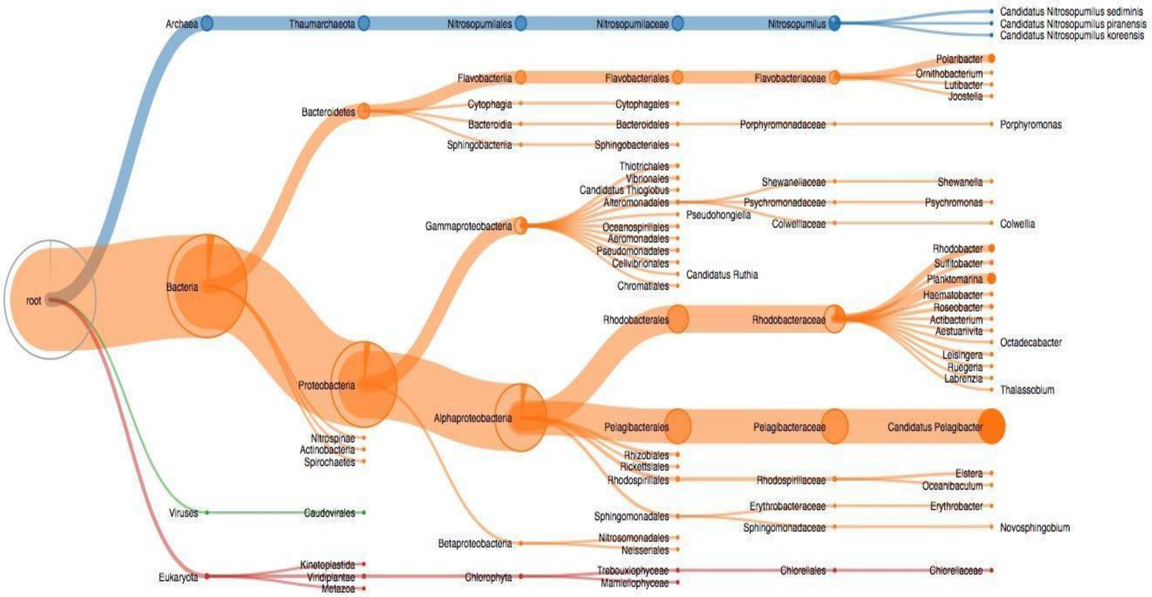

Figure 1: Unipept taxonomy tree

The “Query tabular” and “SQLite to tabular” tools produce a table to generate number of PSMs for each peptide (Steps **14 - 15**) so that a follow up experiment can be performed, if needed.

Parameters for PSMs corresponding to Peptides

```
SELECT sequence as "peptide", count(id) as "PSMs"
FROM bering_psms
WHERE confidence >= 95
GROUP BY sequence
ORDER BY sequence
```

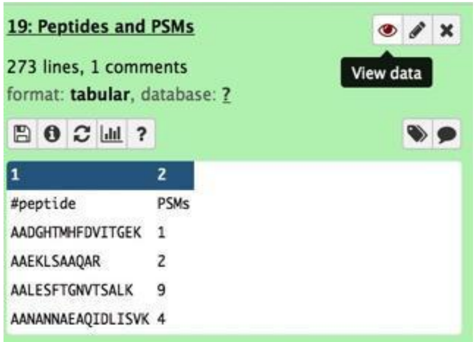

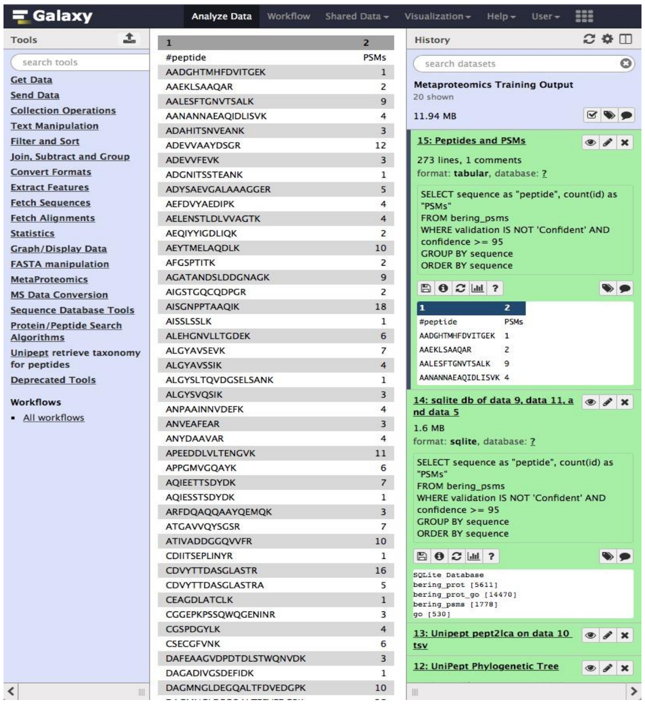

Steps **16** and **17** are the “SQLite to tabular” and “Query tabular” tools to produce a table for each Gene Ontology category summarizing the number of peptides and PSMs associated with each Gene Ontology description.

Parameter for Query Tabular:

SELECT lca.genus,count(psm.sequence) as "PSMs",count(distinct psm.sequence) as "DISTINCT PEPTIDES"
FROM psm LEFT JOIN lca ON psm.sequence = lca.peptide
WHERE confidence >= 95
GROUP BY lca.genus
ORDER BY PSMs desc, 'DISTINCT PEPTIDES' desc

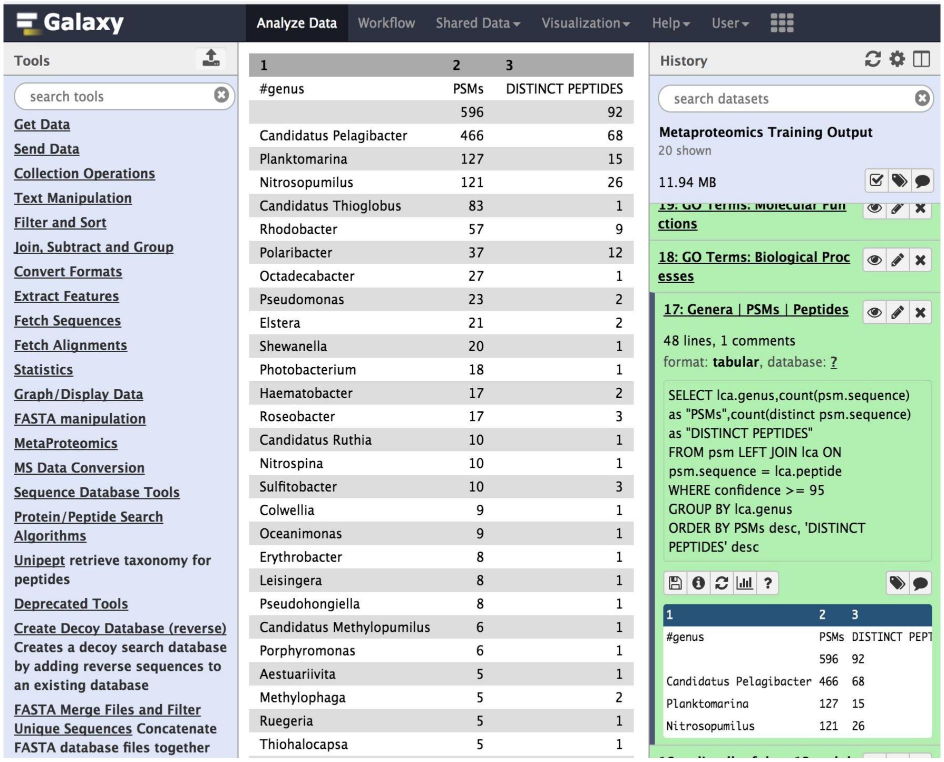

Workflow component: ***Functional analysis***

Steps **11**, **14, 18 - 20** describe the tools used for functional analysis. For Functional analysis, the detected peptide sequences are converted to protein identifications using the Unipept Pept2Pro module.

Parameter used for Unipept Functional Analysis:

Unipept application: pept2prot: UniProt entries containing a given tryptic peptide

Equate isoleucine and leucine: YES
(isoleucine (I) and leucine (L) are equated when matching tryptic peptides to UniProt records)
retrieve extra information: YES
(Return additional information fields: taxon_name, ec_references, go_references, refseq_ids, refdeq_protein_ids, insdc_ids, insdc_protein_ids.

Later, the Query Tabular tool is used to generate, "biological process", "molecular function", and "cellular compartment" GO term outputs along with associated PSMs. For this, the Unipept Pep2Pro output, PSM report and Gene Ontology term (GO) (http://geneontology.org/ontology/go-basic.obo) category annotations are used.

Gene Ontology Categories

a. ***Biological Processes:***

All the peptides and PSMs that matched to a particular Biological Process are listed in the tabular form. **Biological Processes** within Gene Ontology Categories is based on the series or collection of molecular functions.
For example: Translation: *encompasses multiple steps that are involved during the process of translation.*

Parameters used for Determining Biological Processes:

```
SELECT go.description,
count(distinct bering_psms.sequence) as "bering_peptides", count(distinct
bering_psms.id) as "bering_psms"
FROM go JOIN bering_prot_go ON go.go_id = bering_prot_go.go_reference JOIN
bering_prot on bering_prot_go.id = bering_prot.id JOIN bering_psms ON
bering_prot.peptide = bering_psms.sequence
WHERE go.aspect = 'biological_process'
GROUP BY go.description
ORDER BY  bering_peptides desc,bering_psms desc
```

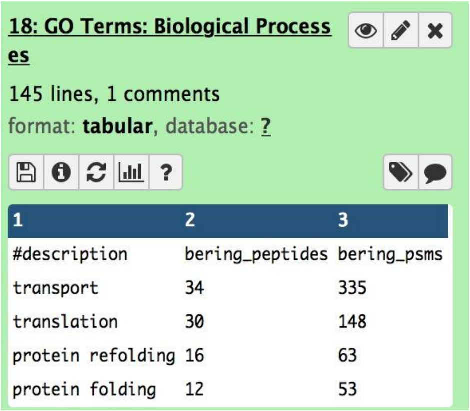

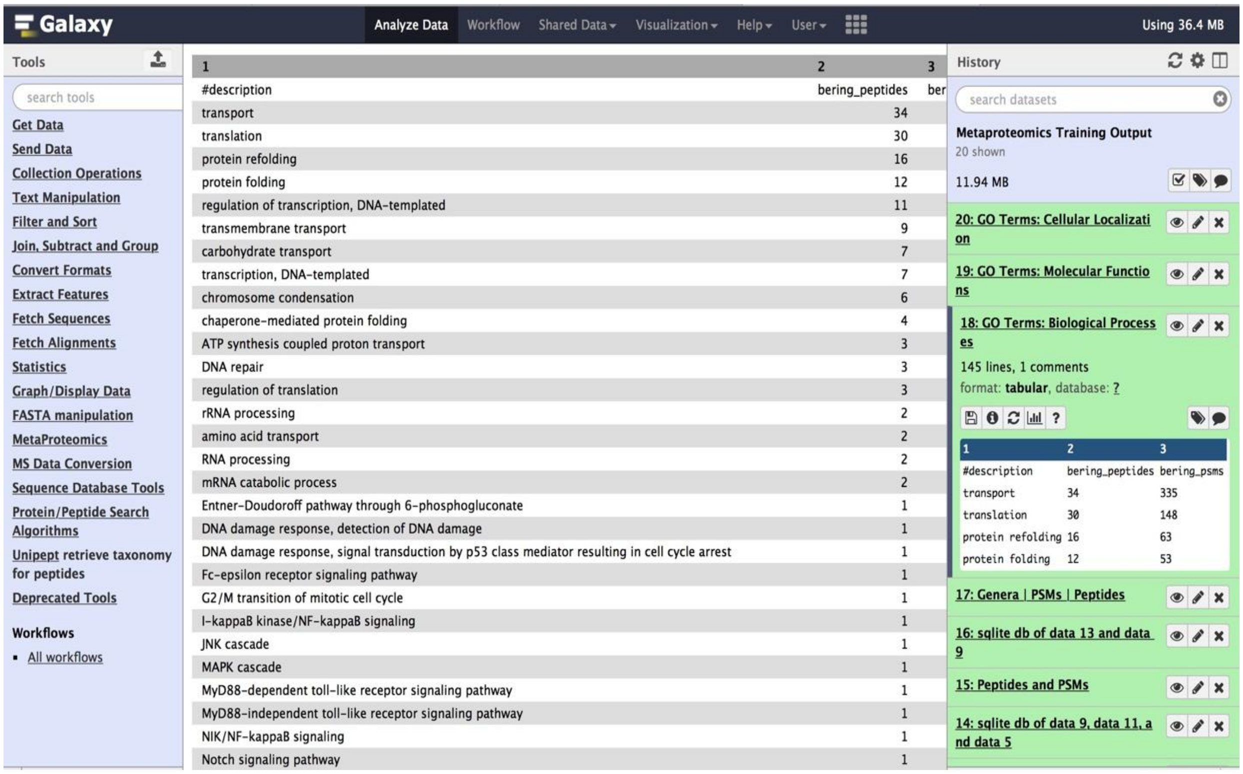

b. Molecular Functions:

In the SQLite to tabular tool, we select all the peptides and PSMs that matched to a particular **Molecular Function** to be listed in the tabular form. **Molecular Functions** category is based on the functions of a gene product.

*For example: **transport activity** is the molecular function of the peptide as its function is to transport molecules.*

Parameters used for Determining Molecular Function:

```
SELECT g.description, count (distinct b.peptide) as "bering_peptides", count (distinct b.id) as
"bering_psms"
FROM go as g JOIN
(SELECT go.description, bering.peptide, bering_psms.id
FROM go LEFT OUTER JOIN bering ON go.go_id = bering.go_reference JOIN bering_psms ON
bering.peptide = bering_psms.sequence
GROUP BY go.description, bering.peptide, bering_psms.id)
as b ON g.description = b.description
WHERE g.aspect = 'molecular_function'
GROUP BY g.description
ORDER BY  bering_peptides desc,bering_psms desc
```

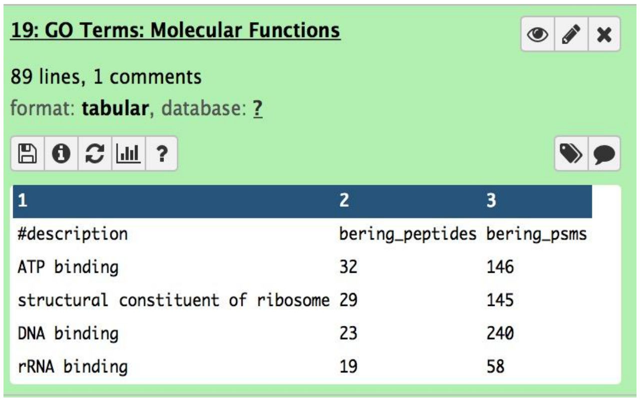

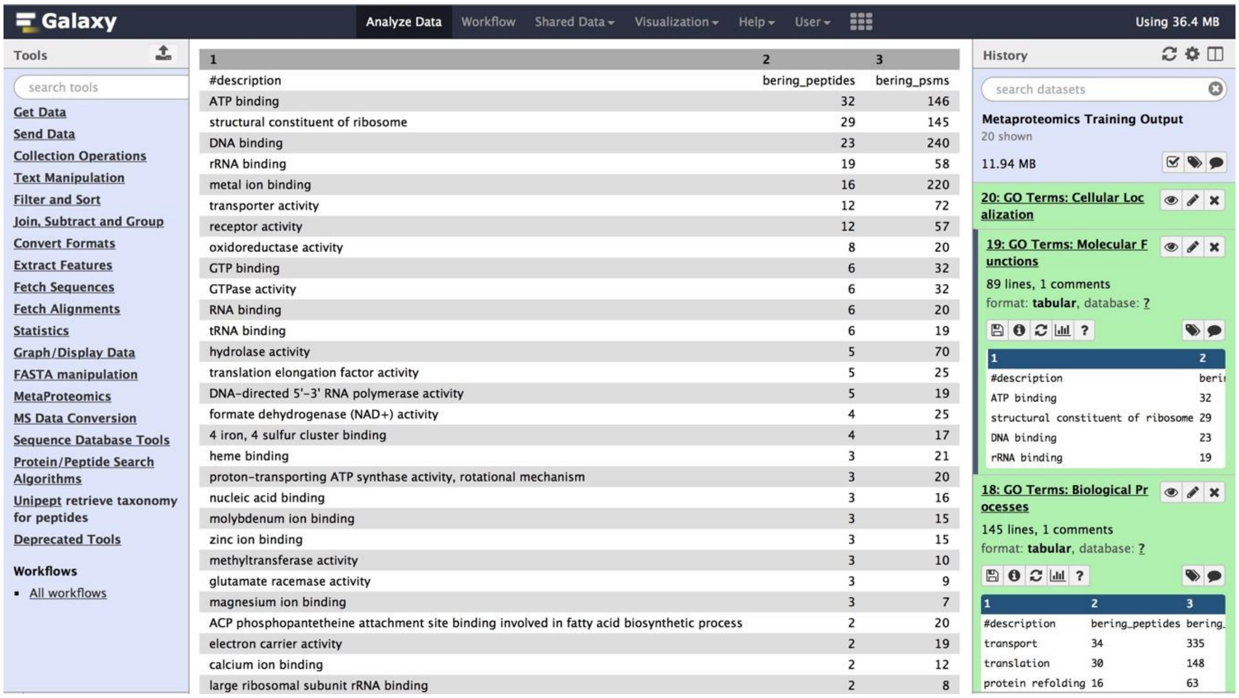

c. Cellular Localization:

In the SQLite to tabular tool, we select all the peptides and PSMs that matched to a particular **Cellular Localization** to be listed in the tabular form. **Cellular Localization** category is based on the location at the levels of subcellular structures and macromolecular complexes.

For example: ***Cytoplasm***

Parameters used for Determining Cellular Localization:

```
SELECT g.description, count(distinct b.peptide) as "bering_peptides", count(distinct b.id) as
"bering_psms"
FROM go as g JOIN
(SELECT go.description, bering.peptide, bering_psms.id
FROM go LEFT OUTER JOIN bering ON go.go_id = bering.go_reference JOIN bering_psms ON
bering.peptide = bering_psms.sequence
GROUP BY go.description, bering.peptide, bering_psms.id)
as b ON g.description = b.description
WHERE g.aspect = 'cellular_component'
GROUP BY g.description
ORDER BY  bering_peptides desc,bering_psms desc
```

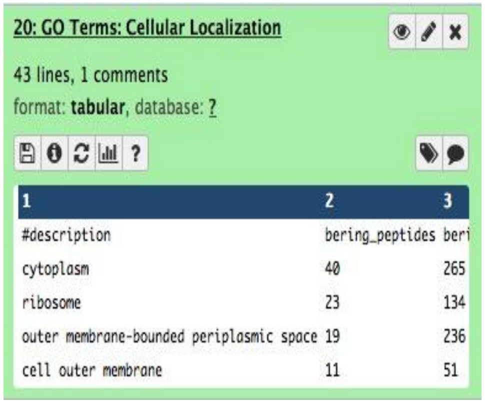

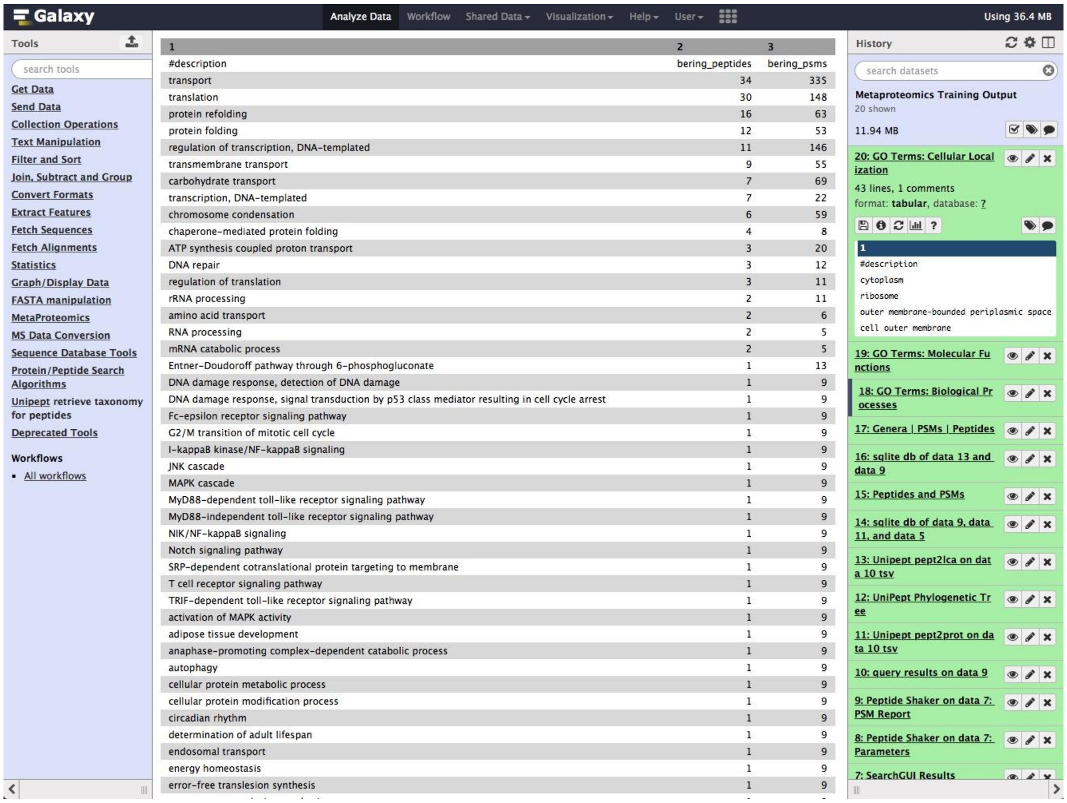

In summary, this single Galaxy workflow takes in MGF input files and searches it against a metaproteomic database to generate PSM report. The PSM report is later used to parse out microbial peptides that are used for taxonomy analysis and functional analysis using Unipept.
